# Efficacy of Intra-arterial Norcantharidin in Suppressing Tumour ^14^C-labelled Glucose Oxidative Metabolism in rat Morris Hepatoma

**DOI:** 10.1155/1996/63403

**Published:** 1996

**Authors:** Peter Mack, Xiao-Fang Ha, Li-Yao Cheng

**Affiliations:** 1 Department of Surgery Singapore General Hospital Outram Road Singapore 0316 Singapore; 2 Department of Biochemistry National University of Singapore 10 Kent Ridge Crescent Singapore 0511 Singapore

## Abstract

Norcantharidin is the demethylated form of Cantharidin, which is the active ingredient of the blister beetle, Mylabris, a long used Chinese traditional medicine. Though not well publicised outside China, Norcantharidin is known to possess significant anti-hepatoma activity, and is relatively free from side effects. In the present study, glucose oxidation in tumour and liver tissue slices harvested from hepatomabearing animals was quantified by measuring the radioactivity of ^14^C-labelled CO released from ^14^C-glucose in oxygen-enriched incubation medium. Results were expressed asa tumour/liver ratio. For comparison, treatments with Norcantharidin, Adriamycin and with hepatic artery ligation were studied. The mean tumour/liver ratio was 4.2+2.2 in untreated controls, but dropped significantly to 2.3+0.5 (p<0.05) with intra-arterial Norcantharidin (0.5 mg/kg) and to 2.3+0.7 (p<0.05) with intra-arterial Adriamycin (2.4 mg/kg), and to 2.2+0.7 (p<0.05) with hepatic artery ligation. However, with intravenous Adriamycin at 2.4 mg/kg, the mean tumour/liver ratio was reduced to only 3.5+2.0 and was not significantly different from untreated controls. It is concluded that intra-arterial Norcantharidin is as effective as intraarterial Adriamycin and hepatic artery ligation in suppressing tumour glucose oxidative metabolism. These results imply that Norcantharidin may have a role to play in the chemotherapy ofprimary livercancer.


*HPB Surgery*, 1996, Vol.10, pp. 65–72
Reprints available directly from the publisher
Photocopying permitted by license only

© 1996 OPA (Overseas Publishers Association)
Amsterdam B.V. Published in The Netherlands
by Harwood Academic Publishers
Printed in Malaysia

### Efficacy of Intra-arterial Norcantharidin in Suppressing Tumour $^{14}C$-labelled Glucose Oxidative Metabolism in rat Morris Hepatoma

PETER MACK*, XIAO-FANG HA* and
LI-YAO CHENG+

*Department of Surgery, Singapore General Hospital, Outram Road,
Singapore 0316, Republic of Singapore
+Department of Biochemistry, National University of Singapore, 10 Kent Ridge Crescent,
Singapore 0511, Republic of Singapore

*(Received 20 September 1995)*

Norcantharidin is the demethylated form of Cantharidin, which is the active ingredient of the blister beetle, Mylabris, a long used Chinese traditional medicine. Though not well publicised outside China, Norcantharidin is known to possess significant anti-hepatoma activity, and is relatively free from side effects. In the present study, glucose oxidation in tumour and liver tissue slices harvested from hepatoma-bearing animals was quantified by measuring the radioactivity of $^{14}C$-labelled $CO_2$ released from $^{14}C$-glucose in oxygen-enriched incubation medium. Results were expressed as a tumour/liver ratio. For comparison, treatments with Norcantharidin, Adriamycin and with hepatic artery ligation were studied. The mean tumour/liver ratio was 4.2±2.2 in untreated controls, but dropped significantly to 2.3±0.5 ($p<0.05$) with intra-arterial Norcantharidin (0.5 mg/kg) and to 2.3±0.7 ($p<0.05$) with intra-arterial Adriamycin (2.4 mg/kg), and to 2.2±0.7 ($p<0.05$) with hepatic artery ligation. However, with intravenous Adriamycin at 2.4 mg/kg, the mean tumour/liver ratio was reduced to only 3.5±2.0 and was not significantly different from untreated controls. It is concluded that intra-arterial Norcantharidin is as effective as intraarterial Adriamycin and hepatic artery ligation in suppressing tumour glucose oxidative metabolism. These results imply that Norcantharidin may have a role to play in the chemotherapy of primary liver cancer.

KEY WORDS: Mylabris Cantharidin liver cancer glucose oxidation Adriamycin hepatic artery ligation

### INTRODUCTION

As searches are underway for new anti-cancer drugs, old drugs are being looked at as novel options to be given by various routes of administration. Although *Mylabris*, the dried body of the Chinese blister beetle, has been used as a traditional medicine in China for more than two thousand years, recent attention is being focused on its anti-cancer properties[1]. The earliest record of Mylabris as a medicine can be tracked back to 300–168 B.C. in China, and later in a medical monograph, *Materia Medica* published in about A.D.77 in Europe[2]. The active constituent of Mylabris has been identified as Cantharidin which possesses not only anti-cancer activity but also causes leukocytosis and haemorrhagic cystitis.

*Correspondence to:* Dr. Peter Mack, Department of Surgery, Singapore General Hospital, Outram Road, Singapore 0316, Republic of Singapore. Fax: 65–2209323 Tel: 65–3214051.

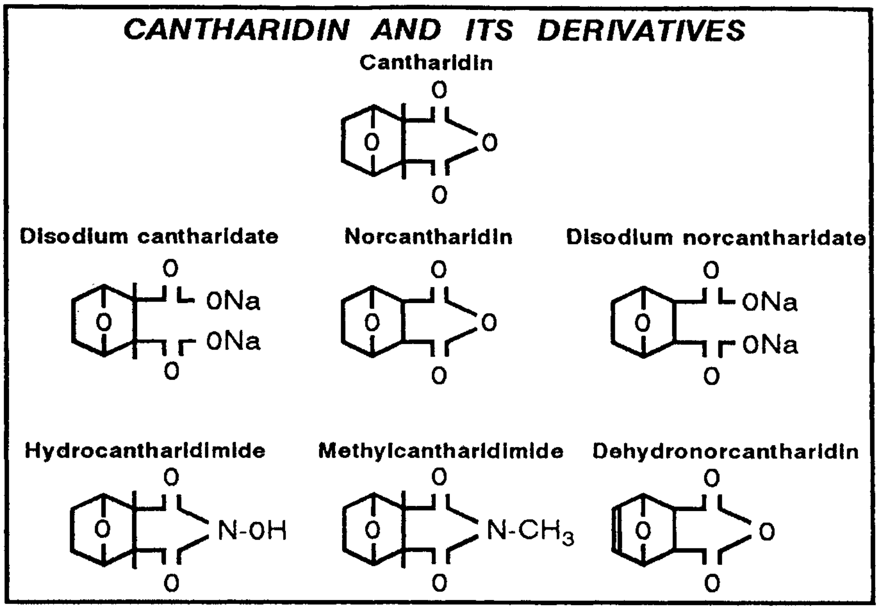

**Figure 1** Chemical structure of Cantharidin and its derivatives.

From this compound, the derivatives of Disodium cantharidate and Norcantharidin (Fig.1) were produced by Wang and his co-workers[2]. These derivatives were in fact more popular than Cantharidin itself, since their anti-hepatoma activities were more potent and yet cause minimal urinary irritation[3]. Amongst them, Norcantharidin has been used in cancer therapy in China since 1984. It has an acute $LD_{50}$ that is 11-fold higher than that of Cantharidin. It also has the highest anti-hepatoma activity among the derivatives, and, in addition, it is free from any depressive effect on the DNA synthesis of bone marrow cells[2].

Many other derivatives of Cantharidin are in existence and have been studied. These include Hydrocantharidimide, Methyl cantharidimide and Dehydronorcantharidin (Fig.1). Most investigations on Cantharidin and its derivatives have, until recently, been carried out within China and reported in their own medical literature[4–7]. As a result for those investigators outside the country, large gaps still exist in their knowledge of these drugs and their efficacy.

At the present state of knowledge, human cancer cell lines that are known to be sensitive to Norcantharidin include HeLa, oesophageal carcinoma (CaEs-17), hepatoma (BEL-7402 and SMMC-7721) and epidermoid laryngocarcinoma (HEP2) cells[2]. Murine tumours, such as Ehrlich ascites carcinoma (EAC) and Hepatoma 22 cells, could be inhibited by Disodium norcantharidate at a dose of 1.0 mg/kg in vivo[2]. Amongst some of the studies done outside China, Yi *et al.* from Australia demonstrated the anti-proliferative effect of Norcantharidin on human myeloid leukaemia cell (K562)[8] While Shimi *et al.* from Egypt showed that Dehydronorcantharidin possesses activity against EAC cells and could prolong the survival of EAC-bearing mice[9].

Despite the scanty data available, there has been a mounting interest in the anti-hepatoma effect of this agent, particularly in China itself mainly because of the high prevalence of primary liver cancer. Hepatocellular carcinoma is endemic in China, and the prognosis of irresectable hepatoma is so poor that any effective chemotherapeutic agent is perceived as a welcome addition to the therapeutic armamentarium. A multicentre clinical trial on the use of Norcantharidinum on 244 patients with advanced primary liver cancer was conducted in 1986 by Wang *et al.* They reported an encouraging response rate of 58.6% with a 39% drop in serum alphafoetoprotein levels. In that study, 37% had regression of hepatomegaly and tumour shrinkage as confirmed with ultrasonography or radioisotope scanning[7].

The present study has been designed to compare quantitatively, the efficacy of Norcantharidin in the treatment of primary liver cancer with the conventional therapeutic options of Adriamycin administration (both intra-arterial and intravenous) and hepatic artery ligation, using an animal hepatoma model. As an indicator of tumour activity, the rate of tumour glucose oxidative metabolism was used. It is long known that most malignant tissues exhibit a high rate of glycolysis[10] and it has also been shown that glucose oxidation in liver tumours is exaggerated compared to normal liver tissue[11,12]. Suppression of overall glucose oxidation has been previously shown to follow the experimental treatment of liver tumours with glucose analogues and by inducing tumour ischaemia[13]. For these reasons it is considered appropriate to choose the suppression of tissue glucose oxidation as an measure of treatment efficacy. As Norcantharidin is already known to be more potent than Adriamycin in the treatment of hepatoma at the same dose level, it was decided to do a comparative in vivo study of the two drugs at recommended clinical dosages (i.e. 0.5. mg/kg for Norcantharidin and 2.4 mg/kg for Adriamycin) rather than at equivalent doses.

### MATERIALS AND METHODS

*Tumour*

The Morris Hepatoma 7777 cell line was used in the present study. The tumour was originally induced by N-2-fluorenylphthalamic acid in inbred Buffalo rats[14]. Morris Hepatoma cells were maintained in monolayer cell culture in Ham's F12 nutrient medium supplemented with 10% calf serum, Penicillin 100 units/ml, Streptomycin 0.1 mg/ml and Amphotericin 2.5 µg/ml (Sigma, Chemical Co., St Louis, MO, USA). After 4 days of incubation at 37°C in 95% air and 5% carbon dioxide, the cells were harvested with 0.25% Trysin (Cytosytems Co., Castle Hill Blue, Australia). Using Trypan Blue (Sigma Chemicals, USA) the cell viability count was performed with a haemocytometer. The chemosensitivity of these hepatoma cells to Adriamycin and Norcantharidin were initially ascertained by in vitro clonogenic survival studies before the planning of the animal experiments.

To establish a solid liver tumour model, a suspension $1.5 \times 10^6$ cells/0.1 ml was injected intra-parenchymally[15] just below the capsule into the left lateral lobe of the rat liver via a small midline laparotomy in the anaesthetised animal. The abdomen was then closed in two layers of continuous suture. A waiting period of 2–3 weeks was needed before the tumour grew into an appropriate size for experiments.

*Animals*

Fifty-five male inbred adult Buffalo rats (NIH, USA) weighing between 250–350 gm were used in this study. All animals were fed a laboratory pellet diet (Wayne Food, UK) and given tap water ad libitum.

*Drugs and Radiochemicals*

Norcantharidin were purchased in glass ampoules of 10mg/2ml from Beijing Fourth Pharmaceutical Works, China. Adriamycin in 10mg vials was purchased from Farmitalia Carlo Erbs Ltd, Herts, Italy. Radioactive labelled D-[UL-$^{14}$C]-glucose (304 mCi/mM) was purchased from Amersham, UK.

*Anaesthesia*

Intramuscular Ketamine at a dose of 7.5 mg/kg was used as anaesthesia for all operative procedures and for all experiments.

*Operative Procedures*

*(1) Gastroduodenal artery cannulation*

Anaesthetised tumour-bearing rats underwent a midline laparotomy to expose the gastroduodenal branch of the common hepatic artery. A fine polyethylene cannula (Portex, 800/100/100/100, UK) filled with normal saline, was inserted under an operating microscope in a retrograde direction into the vessel and secured in position by a 6'O' silk ligature. In one group of tumour-bearing rats ($n$=9), single doses of 0.5 mg/kg of Norcantharidin in 0.3 ml saline were infused over 5 min and the cannula was flushed with a further infusion of 0.5 ml of normal saline. In another group of animals ($n$=8), 2.4 mg/kg of intra-arterial Adriamycin were similarly given. The patency of the hepatic artery one hour after intra-arterial drug administration was confirmed by the presence of pulsations observable under the operating microscope, and all animals were then sacrificed.

*(2) Femoral vein cannulation*

A groin incision was made in the anaesthetised rat, following which the common femoral vein was exposed. Under the operating microscope, a silicone catheter (Dow Corning, Cat No. 602–105, USA) was inserted into its lumen. A dose of Adriamycin at 2.4 mg/kg was infused intravenously through the catheter. All animals were sacriWced one hour later.

*(3) Hepatic artery ligation*

A midline laparotomy was performed in the anaesthetised rat. The falciform ligament and all peritoneal attachments to the liver and the hepatic branch of the hepato-oesophageal artery were divided. The proper hepatic artery, which is the continuation of the common hepatic artery after giving off the gastroduodenal artery, was identified and ligated by a 6'O' silk suture. In the experiment, all animals were sacrified one hour after interruption of hepatic arterial blood flow.

*Glucose Oxidation Measurement*

The method of glucose oxidation measurement, developed by the main author, has been previously described[13]. Tissue samples weighing approximately 20 mg in wet weight were harvested separately, and in turn, from the hepatoma and the adjacent normal liver tissue. From each of these 20 mg samples, 4–5 tissue slices of approximately 0.3 mm thickness were prepared with a sharp scalpel. All the slices of each tissue sample were then incubated in 250 µl of Krebs-Ringer bicarbonate buffer of pH 7.4 which has been supplemented with 100 mg/dl of glucose and gassed with carbogen ($O_2$:$CO_2$=95:5). The incubation medium was placed within centre wells suspended inside glass scintillation counting vials (Kimble, Glass Inc., Vineland, N.J, USA.) from air-tight rubber lined caps. To the medium was added 1 µCi of D-[UL-$^{14}$C] glucose (Amersham, UK). In the outer glass scintillation vial, 500 µl of 1M KOH was placed to trap the $^{14}CO_2$ formed. After 2 hours of incubation in a shaking water bath at 37°C, metabolism was arrested by injecting 50 µl of 1M HCI to the inner vial through the rubber cap. Incubation was then continued overnight at room temperature to allow the $^{14}CO_2$ to be completely trapped in the KOH. Then 10 ml of scintillation fluid (ICN, Cat. No: 882480, USA.) was added to the outer vials and the radioactivity was measured with a Wallac 1410 liquid scintillation counter (Pharmacia,Finland).

In addition to the above, separate liver and tumour specimens approximately 400–500 mg in wet weight were harvested from the sacrificed animal and dried in a hot air oven at 45°C for 3 weeks to obtain the wet wt/dry wt ratio. The results of tumour oxidation of either the tumour or normal liver tissue were then expressed as dpm/mg dry weight.

*Experimental Design*

The study was conducted in two parts:

*Experiment 1*

In the first experiment, 14 rats were used and of these 6 were tumour-bearing animals and 8 were tumour-free animals. A lapse of 3 weeks following tumour cell transplantation was allowed in the tumour-bearing animals on purpose, in order to obtain advanced tumours of sizes up to 8–12 cm$^3$ in volume for the study. All animals, both tumour-free and tumour-bearing, were sacrificed and tissue samples of approximately 20 mg each were obtained separately from the tumour and from the liver tissue. These were assayed for their capacity to oxidise $^{14}$C-glucose as described in the preivous section. All results were expressed as dpm/mg dry wt. A comparison was made between the glucose oxidation of hepatoma and that of liver tissue in the same tumour-bearing animals. A second comparison was made in the glucose oxidation between tumour-bearing liver and tumour-free liver in the two respective groups of animals.

*Experiment 2*

Forty-one tumour-bearing rats were used in this experiment and all animals had small-sized hepatomas. A lapse of 10–14 days after tumour cell inoculation in the animals was allowed so that the tumour sizes at the time of experiment were each approximately 1–2 cm$^3$. in volume. The objective of using small tumours in this study design was two-fold. Firstly, the associated operative difficulties of performing hepatic artery ligation and gastroduodenal artery cannulation in the presence of bulky liver tumour could be avoided. Secondly, the design permits a comparison between the tumour/liver ratio of glucose oxidation of small tumours in this group against that of large advanced tumours in the Experiment 1.

The 41 tumour-bearing animals in this experiment were assigned to 5 groups as follows:

(1) Control group ($n$=9), in which no treatment was given.

(2) Intravenous Adriamycin group ($n$=7), in which Adriamycin was infused as a single dose of 2.4 mg/kg in 0.3 ml saline over 5 minutes through the femoral vein, followed by flushing with 0.5 ml of saline.

(3) Intra-arterial Adriamycin group ($n$=8), in which Adriamycin was infused as a single dose of 2.4 mg/kg in 0.3 ml saline over 5 minutes through the gastroduodenal artery followed by flushing with 0.5 ml of saline.

(4) Intra-arterial Norcantharidin group ($n$=9), in which Norcantharidin was infused as a single dose of 0.5 mg/kg (recommended dose) in 0.3 ml saline over 5 minutes, followed by flushing with 0.5 ml of saline.

(5) Hepatic artery ligation group ($n$=8), in which all animals had their proper hepatic artery ligated in the manner described above.

All animals in the control group were sacrificed untreated whereas those in the other four treated groups were sacrificed one hour after institution of treatment. Tisssue specimens were separately harvested from the hepatoma and adjacent normal liver tissue and were assayed for their glucose oxidation capacity. The results of glucose oxidation of the hepatoma tisssue and of the normal liver tissue in each animal was subsequently expressed as "tumour/liver ratio" in that particular animal.

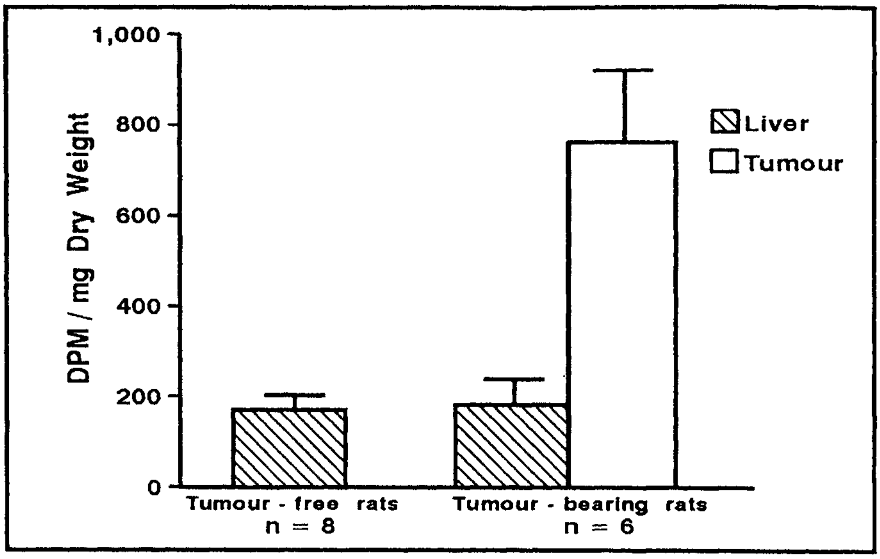

**Figure 2** $^{14}$C-glucose oxidation in hepatoma-bearing (n=6) and in tumour-free rats (n=8) expressed as dpm/mg dry wt. Vertical lines on top of bars indicate standard deviations of means. The glucose oxidation of hepatoma tissue was 4.3 times as high as the adjacent liver parenchyma (p<0.01). No significant difference exists between the liver of tumour-bearing and the liver of tumour-free rats.

*Statistics*

All data were expressed as mean ± standard deviation. The Student's t-test and the Analysis of Variance with Duncan's multiple range test were used for statistical evaluation for differences between groups. Confidence limits for statistically significant differences were expressed at the 5% and 1% levels.

### RESULTS

In the first experiment, the mean glucose oxidation of liver tissue was 168.6±34 dpm/mg dry wt. in the tumour-free rats and was 183.5±53 dpm/mg dry wt. in the tumour-bearing animals. They were not significantly different from one another. The hepatoma tissue in the tumour-bearing rats however showed a glucose oxidation of 763.6±167 dpm/mg dry wt. and was markedly higher than their corresponding normal liver tissue ($p<0.01$) (Fig. 2). The mean tumour/liver ratio was 4.3±1.1 in this group of advanced liver tumours.

In the second experiment, mean tumour/liver ratio in the untreated animals in the control group with small hepatomas was 4.2±2.2. This result was very close to the ratio obtained with the advanced tumours in the first experiment. One hour after treatment with intra-arterial Adriamycin (2.4 mg/kg), this tumour/liver ratio was found to drop significantly to 2.3±0.7 ($p<0.05$). Similar significant reductions of this tumour/liver ratio to 2.3±0.5 ($p<0.05$) was found one hour after hepatic artery ligation. However, when Adriamycin was given intravenously in a dosage identical to that of the intra-arterial Adriamycin group (i.e. 2.4 mg/kg), the tumour/liver ratio decreased slightly after one hour to only 3.5±2.0 and this was not significantly different from that of untreated controls (Fig. 3).

### DISCUSSION

Mylabris is potentially attractive as a chemotherapeutic drug because it possesses anti-cancer properties and at the same time does not

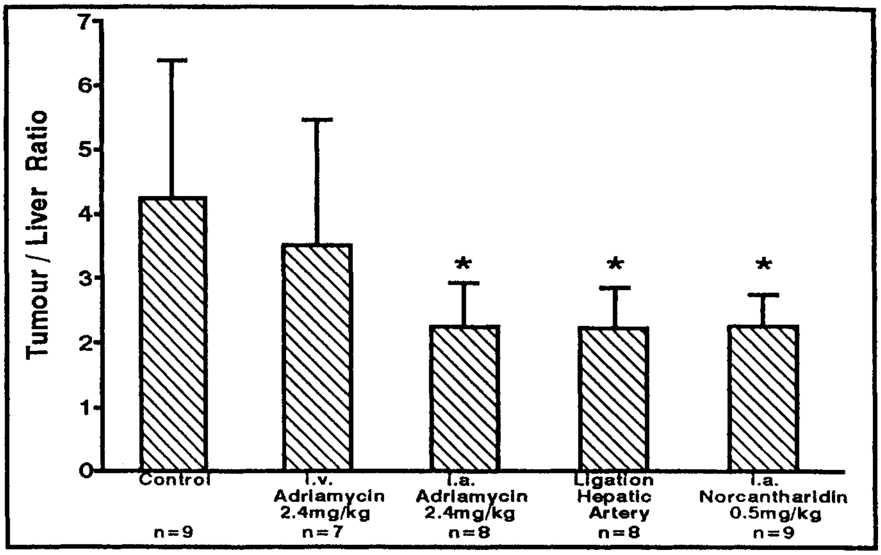

**Figure 3** Tumour/liver ratio of glucose oxidation in various treatment groups: Controls ($n$=9), i.v. Adriamycin ($n$=7), i.a. Adriamycin ($n$=8), Hepatic artery ligation ($n$=8), and i.a. Norcantharidin ($n$=9). *indicates statistical significance ($p$<0.05) when compared with controls. Vertical lines on top of bars indicate standard deviations of means.

inhibit bone marrow cells. Its active constituent, Cantharidin, has already been synthesised but the latter compound has been found to irritate the skin and the urinary bladder strongly. Disodium cantharidate was subsequently prepared instead in an effort to lower the intrinsic toxicity of Cantharidin. Then it was realised that further reduction in toxicity can be achieved by preparing the demethylated form, Norcantharidin, without compromising on the anti-tumour activity. In fact, this latest derivative has been found to possess even more potent anti-hepatoma effects than all other derivatives. In one study where 285 hepatoma patients were treated by Norcantharidin, the mean survival time was 11.1 months and the 1-year survival was 30%. This compared favourably against a mean survival of 4.7 months and a 1-year survival of 17% in another group of 102 cases treated with other conventional chemotherapeutic agents including 5-fluorouracil, hydroxy-camptothecine, thiophosphoramide, vincristine and mitomycin[2].

Not much is known about the mechanism of action of Norcantharidin or its derivatives with respects to their anti-cancer activity. In one study, exposure of K562 cells to Norcantharidin resulted in blocking of the cells in G2+M phase of the cell cycle[8]. In another study, the incorporation of thymidine and uridine were affected showing that the DNA and RNA synthesis in EAC-cells was significantly decreased by the administration of Dehydronorcantharidin[9]. A recent study of Norcantharidin on normal and malignant haemopietic cells has provided some new insight into the cellular and molecular actions of this compound[16]. It showed that Norcantharidin stimulated the cell cycle progression of granulocyte-macrophage colony-forming cells, stimulated DNA synthesis and increased the frequency of mitotic cells in short-term human bone marrow cultures. It also stimulated the human bone marrow cultures. It also stimulated the production of interleukin-1β, colony stimulating activity and tumour necrosis factor-α. Continuous in vitro Norcantharidin treatment however, inhibited both DNA synthesis and granulo-cyte-macrophage colony forming cell growth. Low doses of Norcantharidin in HL-60, K-562 leukaemic and MRC5V2 fibroblast cell lines accelerated the $G_1$-S phase transition while higher doses or prolonged incubations inhibited the cell cycle at the $G_2$/M phases or during the formation of postmitotic daughter cells. Norcantharidin also impaired the neogenesis of chromatin material and nuclear membrane during the M/$G_1$ phase transition in K-562 cells. These effects of Norcantharidin may be due to its inhibitory effect on protein phosphatase type 2A activity which regulates the cell cycle and may partly explain its anti-cancer activity.

The development of a useful anti-cancer drug outside the West with minimal documentation in the English medical literature often invites reluctance on the part of clinicians to adopt its usage. This typically applies to drugs developed from Chinese traditional medicine. In such situations it is not uncommon that a certain amount of laboratory evaluation to be repeated or extended in various forms before the scientific community can come forward with some concensus on its acceptablility. In the case of Norcanthardin, which appears attractive in its use in the treatment of primary liver cancer, the authors were inspired to evaluate its efficacy quantitatively in an experimental setting. While suppression of glucose oxidative metabolism was used in this experiment because it is a quick and convenient parameter for short term efficacy, further experiments showing its effect on tumour burden at various time points following drug administration will eventually be necessary to generate more enthusiasm in using the drug. Although the scarcity of relevant literature in English might be regarded by some as a negative aspect in evaluating studies of this nature, the same may be used to argue in favour of the initiation of such studies.

The metabolic measurements in this study confirmed that the Morris Hepatoma used is characterised by high metabolic activity and is 4.2 times as active as that of liver tissue in terms of glucose oxidation. In addition, this ratio does not appear to differ between early and advanced hepatomas. By comparison, a tumour/liver ratio of 10.5 was noted in m'-methyl-*p*-dimethylaminoazobenzene induced hepatoma in Sprague-Dawley (Hisaw) strain rats[12] and a ratio of 2.8 found in transplanted colonic adenocarcinoma in the liver in Wistar-Furth rats[13]. It has been previously shown that a growing malignant tumour behaves as a trap for glucose and acts as a powerful hypoglycaemic factor imposing a strain on the host to maintain glucose homeostasis[17]. The liver tissue of the tumour-bearing host, which is the site for glucostatic functions, might be expected to mainfest disturbances in glucose metabolism. However, no differences in glucose oxidation have been observed between the liver tissue of tumour-free and that of tumour-bearing rats in this study.

Following tumour treatment with intra-arterial Norcantharidin, the tumour/liver ratio was nearly halved at the end of the first hour. A similar effect was also observed when the tumour was treated with intra-arterial Adriamycin, indicating that both drugs, when given by the arterial route, exhibit a similar potency. When the same dosage of Adriamycin was given by the intravenous route, the depression in tumour metabolism was significantly less. This can be explained by the fact that the concentration of drug delivered by the intravenous route to the tumour site is significantly less when compared to that delivered by the arterial route[18]. In one study, when the hepatic venous drug concentrations were compared between administration by the intra-venous and the intra-arterial routes, up to fivefold higher concentration is found when given by the hepatic artery route[19].

The results of the present study concurs with current clinical recommendations that chemotherapy for primary liver cancer, if indicated, should preferably be administered by the arterial route[20]. The results of intravenous Adriamycin, even though it does not contribute to the comparative assessment of the efficacy of intra-arterial Norcantharidin, has nonetheless been included in this study mainly because, it remains a treatment modality that is commonly used clinically for irresectable hepatoma. The message must be reinforced to the clinical oncologist that the intravenous route should, whenever feasible, be discarded in favour of the intra-arterial route in the administration of chemotherapy for primary liver cancer.

It is known that the blood flow to liver tumours are predominantly supplied by the arterial route[21,22] and it has been shown that ligation of hepatic artery could inhibit liver tumour growth[23,24,25], at least in the initial stages before collaterals developed[26]. It is presumed that the inhibition in tumour growth by ischae mia is mediated through suppression of tumour metabolism. Since hepatic artery ligation is clinically an effective, though temporary, method of treating irresectable liver tumours that yields consistent results, its inclusion as a study group for comparison has provided a good yardstick on which to evaluate the efficacy of all other forms of treatment. Hepatic artery ligation has been shown to lower the hepatoma glucose oxidation capacity by as much as 48% after one hour in this study. By comparison, only a 25% reduction in glucose oxidation was achievable after one hour following hepatic artery ligation in a metastatic liver adenocarcinoma model in a similar previous study[13].

In conclusion, Norcantharidin is attracting interest from clinicians because of its anti-hepatoma properties. When given by the intra-arterial route, it is as effective as intra-arterial Adriamycin and hepatic artery ligation in the suppression of tumour oxidative glucose metabolism in a hepatocellular carcinoma model in the rat. As this drug has minimal side effects and does not cause bone marrow suppression, it has an edge over Adriamycin and may potentially establish a place in the chemotherapy of primary liver cancer in future.

### ACKNOWLEDGEMENTS

The authors wish to thank: (1) Professor Yu Zhu Yuan of the Liver Cancer Institute of Shanghai Medical University, China, for introducing to them the use of Norcantharidin in the treatment of primary liver cancer, (2) Mr.Robert Ng and Ms. Song In Chin of the Department of Experimental Surgery, Singapore General Hospital, for their technical assistance in conducting this study, and (3) Professor Wang Guang-Sheng of Beijing Fourth Pharmaceutical Works, China, for sharing with us his expert knowledge on Norcanthardin, and for his valuable comments on this manuscript.

This project was supported by a grant from the Singapore Totalisator Board.

### REFERENCES

1. Wang, G.S. (1980) Antitumor studies of mylabris and its development. (Chinese) *Chinese Pharmaceutical Bulletin.*, **3**, 23–26.
2. Wang, G.S. (1989) Medical uses of mylabris in ancient China and recent studies. *J.Ethnopharmacol.*, **26**, 147–162.
3. Wang, G.S. (1983) Hydrolysis and demethylation of cantharidin on the relief of its urinary irritation. (Chinese). *Chinese Pharmaceutical Bulletin.*, **18(7)**, 18–19.
4. Wang, G.S. (1976) Pharmacology and clinical trials of mylabris and camptotheca acuminata. (Chinese). *Beijing Research Information on Prevention and Treatment of Tumors .*, **1**, 60–62.
5. Chen, R.T., Hua, Z., Yang, J.L. *et.al.* (1977) Studies on antitumor actions of cantharidin. (Chinese) *National Medical Journal of China.*, **57(8)**, 475–48.
6. Dong, C., Cheng, F.H., Jin, T.Y., Li, B.M. (1982) Observations on the antitumor effect with norcantharidin in vitro. (Chinese) *Yixue Ziliao (Health Bureau of Fuzhou Military Region).*, **1**, 52–53.
7. Wang, G.S., Huang, J.K., Lu, F.X., *et al.* (1986) Results of clinical trials in 244 cases of primary hepatoma with norcantharidin. (Chinese). *Chinese Pharmaceutical Bulletin.*, **21(2)**, 90–93.
8. Yi, S.N., Wass, J., Vincent, P., Iland, H.(1991) Inhibitory effect of norcantharidin on K562 human myeloid leukaemia cells in vitro. *Leuk.Res.*, **15**, 883–886.
9. Shimi, I.R., Zaki, Z., Shoukry, S., Medhat, A.M. (1982) A new antitumor substance, 7-oxabicyclo(2.2.1)-5-heptene-2, 3-dicarboxylic anhydride. *Eur. J. Cancer Clin. Oncol.*, **18**, 785–793.
10. Warburg, O. (1930) *The metabolism of tumours*. London: Constable & Co.
11. Zamecnik, P.C., Loftfield, R.B., Stephenson, M.L., Steele, J.M. (1951) Studies on the carbohydrate and protein metabolism of the rat hepatoma. *Cancer Res.*, **11**, 592–602.
12. Olson, R.E. (1951) Oxidation of $C^{14}$ -labelled carbohydrate intermediates in tumor and normal tissue. *Cancer Res.*, **11**, 571–584.
13. Mack, P., Ahren, B., Jeppsson, B., Zuxing, K., Bengmark, S. (1988) Influence of 2-Deoxy-D-glucose and arterial ischaemia on glucose oxidative metabolism and growth of liver cancer *Eur. J. Cancer Clin. Oncol.*, **24**, 1433–1437.
14. Morris, H.P., Dyer, H.M., Wager, B.P., Miyaji, H., Rechcigi, M.Jr. (1964) Some aspects of the development, biology and biochemistry of rat hepatomas of different growth rate. *Adv. Enzyme Regul.*, **2**, 321–333.
15. Yang, R., Rescorla, F.J., Reilly, C.R., Faught, P.R., Sanghvi, N.T., Lumeng, L., Franklin, T.D., and Grosfeld, J.L. (1992) A reproducible rat liver cancer model for experimental therapy: Introducing a technique of intrahepatic tumor transplantation. *J.Surg. Res.*, **52**, 193–198.
16. Liu, X.H., Blazsek, M.C., Legras, S., Marion, S., Quittet, P., Anjo, A., Wang, G.S., Misset, J.L. (1995) Effects of norcantharidin, a protein phosphatase type-2A inhibitor, on the growth of normal and malignant haemopoietic cells. *Eur. J. Cancer.* (In press).
17. Shapot, V.S.(1972) Some biochemical aspects of the relationship between the tumor and the host. *Adv. Cancer Res.*, **15**, 253–286.
18. Erichsén, C., Christenson, P.I., Eriksson, G., Yngner, T., Jönsson, P.E., Stenram, U. (1986) Effects of administration routes on the uptake of uridine and 5-fluorouridine into an adenocarcinoma transplated to rat liver. *J Surg Oncol.*, **33**, 76–80.
19. Ensminger, W.D., Rosowsky, A., Raso, V. *et al.* (1987) A clinical-pharmacological evaluation of hepatic arterial infusions of 5-fluoro-2'-deoxyuridine and 5-fluorouracil. *Cancer Res.*, **38**, 3784–3792.
20. Fortner, J.G., Mulcare, R.J., Solis, A., Watson, R.C., Golbey, R.B. (1973) Treatment of primary and secondary liver cancer by hepatic artery ligation and infusion chemotherapy. *Ann. Surg.*, **178**, 162–172.
21. Breedis, C., Young G. (1954) The blood supply of neoplasms in liver. *Am. J. Pathol.*, **30**, 969–985.
22. Ackerman, N.B., Lien, W.M., Kondi, E.S., Silverman, N.A. (1969) The blood supply of experimental liver metastases. I. The distribution of hepatic artery and portal vein blood to "small" and "large" tumors. *Surgery*, **66**, 1067–1072.
23. Nilsson, L.A.V., Zettergren, L. (1967) Effect of hepatic artery ligation on induced primary liver carcinoma in rats. *Acta. Path. et Microbiol. Scandinav.*, **71**, 187–193.
24. Nilsson, L.A.V. (1966) Therapeutic hepatic artery ligation in patients with secondary liver tumors. *Rev. Surg.*, **23**, 374–376.
25. Balasegaram, M. (1972) Complete hepatic dearterialization for primary carcinoma of the liver. *Amer. J. Surg.*, **124**, 340–345.
26. Koehler, R.E., Korobkin, M., Lewis. F.(1975) Arteriographic demonstration of collateral arterial supply to the liver after hepatic artery ligation. *Radiology*, **117**, 49–54.

